# Apremilast Microemulsion as Topical Therapy for Local Inflammation: Design, Characterization and Efficacy Evaluation

**DOI:** 10.3390/ph13120484

**Published:** 2020-12-21

**Authors:** Paulo Sarango-Granda, Marcelle Silva-Abreu, Ana Cristina Calpena, Lyda Halbaut, María-José Fábrega, María J. Rodríguez-Lagunas, Natalia Díaz-Garrido, Josefa Badia, Lupe Carolina Espinoza

**Affiliations:** 1 Department of Pharmacy, Pharmaceutical Technology and Physical Chemistry, Faculty of Pharmacy and Food Sciences, University of Barcelona, 08028 Barcelona, Spain; paulogranda92@gmail.com (P.S.-G.); marcellesabreu@gmail.com (M.S.-A.); halbaut@ub.edu (L.H.); lcespinoza@utpl.edu.ec (L.C.E.); 2 Institute of Nanoscience and Nanotechnology (IN2UB), University of Barcelona, 08028 Barcelona, Spain; 3 Department of Experimental and Health Sciences, Parc de Recerca Biomèdica de Barcelona, University Pompeu Fabra (UPF), 08005 Barcelona, Spain; mjfabrega.f@gmail.com; 4 Department of Biochemistry and Physiology, Faculty of Pharmacy and Food Sciences, University of Barcelona, 08028 Barcelona, Spain; mjrodriguez@ub.edu (M.J.R.-L.); natalia.diaz.garrido@gmail.com (N.D.-G.); josefabadia@ub.edu (J.B.); 5 Nutrition and Food Safety Research Institute (INSA-UB), 08921 Santa Coloma de Gramenet, Spain; 6 Institute of Biomedicine of the University of Barcelona (IBUB), Sant Joan de Déu Research Institute, 08028 Barcelona, Spain; 7 Departamento de Química y Ciencias Exactas, Universidad Técnica Particular de Loja, Loja 1101608, Ecuador

**Keywords:** apremilast, phosphodiesterase 4, microemulsion, skin diseases, inflammation

## Abstract

Apremilast (APR) is a selective phosphodiesterase 4 inhibitor administered orally in the treatment of moderate-to-severe plaque psoriasis and active psoriatic arthritis. The low solubility and permeability of this drug hinder its dermal administration. The purpose of this study was to design and characterize an apremilast-loaded microemulsion (APR-ME) as topical therapy for local skin inflammation. Its composition was determined using pseudo-ternary diagrams. Physical, chemical and biopharmaceutical characterization were performed. Stability of this formulation was studied for 90 days. Tolerability of APR-ME was evaluated in healthy volunteers while its anti-inflammatory potential was studied using in vitro and in vivo models. A homogeneous formulation with Newtonian behavior and droplets of nanometric size and spherical shape was obtained. APR-ME released the incorporated drug following a first-order kinetic and facilitated drug retention into the skin, ensuring a local effect. Anti-inflammatory potential was observed for its ability to decrease the production of IL-6 and IL-8 in the in vitro model. This effect was confirmed in the in vivo model histologically by reduction in infiltration of inflammatory cells and immunologically by decrease of inflammatory cytokines IL-8, IL-17A and TNFα. Consequently, these results suggest that this formulation could be used as an attractive topical treatment for skin inflammation.

## 1. Introduction

**Figure 1:**
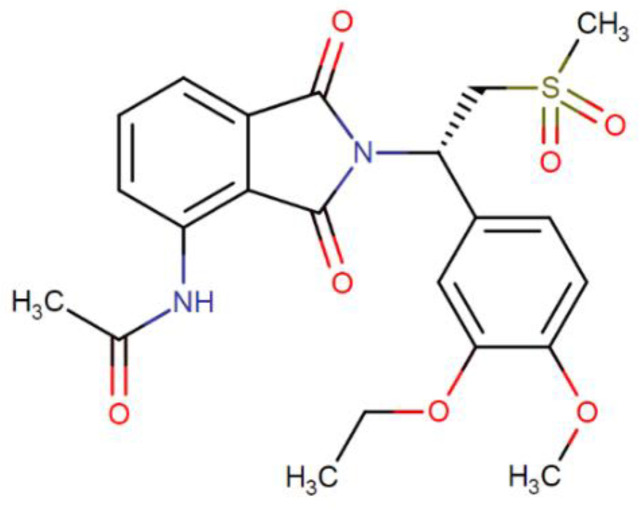
Apremilast chemistry structure.

Inflammation constitutes a defense mechanism of the body. It is the response of the immune system against numerous harmful stimuli, among which include pathogens, toxic compounds and damaged cells [1]. However, inappropriate or excessive inflammatory responses can trigger chronic inflammation implicated in the pathogenesis of a wide variety of skin disorders [2]. The skin inflammatory response is mediated by cytokine secretions in response to injury, including tumor necrosis factor-alpha (TNF-α) and interleukin (IL) -6, IL-8 and IL-17. Therapeutic treatment involves treating symptoms by interrupting the inflammatory process [3]. The mechanism based on phosphodiesterase 4 (PDE4) enzyme inhibition has been used for the treatment of inflammatory and autoimmune diseases. PDE4 is one of the many classes of phosphodiesterase enzymes (PDE) that participate in the hydrolysis of cAMP [4]. PDE4 inhibition causes a decrease in the expression of pro-inflammatory cytokines, such as interleukin (IL)-17 and tumor necrosis factor alpha (TNF-α), and an increase in anti-inflammatory mediators, such as IL-10 [5]. Apremilast (APR) is a selective PDE4 inhibitor chemically identified as *N*-{2-[(1S)-1-(3-ethoxy-4-methoxyphenyl)-2-(methylsulfonyl) ethyl]-2,3-dihydro-1,3-dioxo-1*H*-isoindole-4-yl} acetamide. It is a small and versatile molecule whose formula and molecular weight are C_22_H_24_N_2_O_7_S and 460.5 g/mol, respectively [6,7]. Figure 1 shows the chemical structure of APR [8]. APR was approved in 2014 by the United States Food and Drug Administration (FDA) as oral therapy for the treatment of moderate-to-severe plaque psoriasis and active psoriatic arthritis [9,10]. This drug causes an intracellular accumulation of cyclic adenosine monophosphate (cAMP), resulting in a modification in the signaling pathways of cells belonging to the innate (monocytes) and adaptive (T cells) immune system as well as non-immune cells (keratinocytes) [11,12]. In the last decade, novel drug delivery systems for APR has been reported in order to improve its solubility and bioavailability including PLGA nanoparticles [13], amorphous solid dispersion [14], nanostructured lipid carriers [15] and nanocrystal-based formulations [16]. Currently, APR is available in tablet form of 10, 20 and 30 mg for oral administration [17]. However, this route of administration presents notable disadvantages related with adverse effects, first-pass metabolism and, moreover, is not suitable for patients with swallowing difficulties. In particular, topical therapy targeting a specific inflammatory mediator on the skin promises a local pharmacological effect with fewer side effects [18]. This route represents a convenient and painless alternative in the treatment of dermatological diseases because it allows drugs to be directed to the affected sites within the skin [19]. However, the main obstacle of topical formulations is to overcome the stratum corneum (SC), which limits the speed of transdermal diffusion of various drugs to achieve the intended therapeutic effect [20,21,22]. Drug permeation through the skin depends on the physicochemical characteristics of the drug as well as the chemical composition and physical properties of the carrier. APR is categorized as a class IV drug, according to the Biopharmaceutical Classification System (BCS), due to its low solubility and low permeability [23]. Hence, the incorporation of APR into nanotechnology-based drug delivery systems such as microemulsions (MEs) could be used as a strategy to improve its solubility and permeability in order to improve dermal bioavailability and consequently to achieve local anti-inflammatory efficacy [24]. MEs have been proposed as promising nanocarriers to deliver anti-inflammatory drugs in the skin such as cyclosporine, methotrexate and tacrolimus due to their capability to solubilize highly hydrophobic drugs as well as their capability to enhance drug uptake into the skin [25,26,27].

MEs are thermodynamically stable formulations characterized by being transparent, monophasic optically isotropic systems. They are formed by adequate proportions of water, oil, surfactant and co-surfactant, and usually show small droplets that range from 10 to 100 nm [28]. MEs have a high interfacial stability that optimize the stability of the formulation. Among the benefits offered by this type of system include reduced droplet size and greater solubility, especially for poorly soluble and unstable drugs [29,30]. In addition, due to the nature of the components that are in the formulation, in some cases they can serve as a penetration enhancer, thereby increasing the rate of penetration of the drug through the skin.

Considering that there are currently no complete or relevant studies of drug delivery systems for APR by topical application, the purpose of this study was to design and characterize an APR-ME as a strategy to improve the solubility and permeability of APR in order to provide a topical therapy alternative with a local anti-inflammatory effect on the skin.

## 2. Results

### 2.1. Validation of the Analytical Method

The results obtained related to the analytical method can be found in Supplementary Material. The linearity was evaluated from three calibration curves within a range of 1.25 to 200 µg/mL (Table S1). No point was left in the calibration during the validation and the data showed good linearity of the proposed method (Figure S1). The value of *r*^2^ in each of the calibration curves was above 0.999, thus indicating a linear relationship between analyte concentration and peak area. No statistically significant differences were found after the one-way analysis of variance (one-way ANOVA) test of the calibration curves, where *p* value = 0.91.

The precision and accuracy values were obtained from APR standard concentration ranged from 1.25 to 200 µg/mL. The inter-day precision and accuracy were calculated after analyzing the samples on three different days. The results were reported in Tables S2 and S3. These results showed good precision with values of RSD below 3.77%, while accuracy was shown to have a maximum RE of −8.36% for lowest standard concentration.

Measuring robustness allows recognition of the effect of operational parameters on results and provides an indication of applicability during the study. This parameter was determined by evaluating the retention time of the asset with the variations of concentration change of the mobile phase shown in Table S4.

Specificity (Figure S2) was determined by analysis of the blank mobile phase control, the 100 µg/mL standard, the skin blank as a control, and APR extracted from human skin after permeation study. No interference on the chromatogram with respect to retention time of APR was observed.

The LOD and LOQ were calculated using the standard deviation of response and the slope of the calibration curve from 1.25 to 200 µg/mL, described in Section 4.2. From the flow and the Y-intercept of the three straight lines (Table S5), the LOD for APR was set at 1.13 ± 1.04 µg/mL and LOQ at 3.42 ± 3.16 µg/mL. These results indicate that the method is sensitive enough for the APR determination.

### 2.2. Pseudo-Ternary Phase Diagrams and APR-ME Preparation

**Figure 2:**
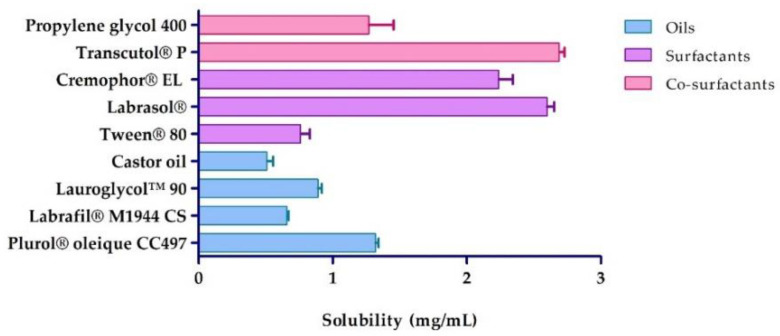
Apremilast solubility in different vehicles (n = 3).

The APR solubility at 25 °C in the different assayed oils, surfactants and co-surfactants are shown in Figure 2. The components that showed the greatest capacity to solubilize the drug were selected as constituents of APR-ME. In this study, Plurol^®^ oleique CC497 (oil, solubility 1.32 ± 0.04 mg/mL), Labrasol^®^ (surfactant, solubility 2.60 ± 0.09 mg/mL) and Transcutol^®^ P (co-surfactant, solubility 2.69 ± 0.07 mg/mL) were used.

**Figure 3:**
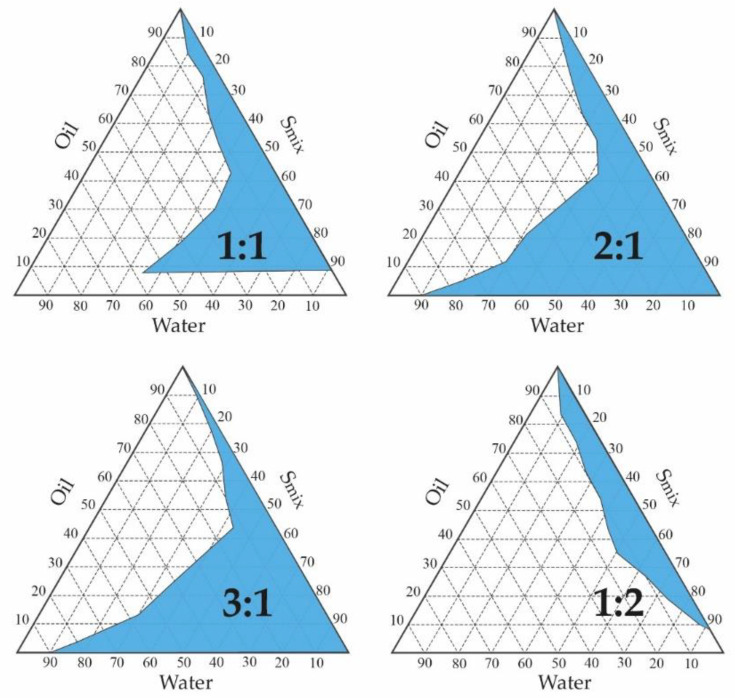
Pseudo-ternary phase diagrams using different ratios of surfactant/co-surfactants (S_mix_) at 25 °C.

Four pseudoternary phase diagrams were performed to establish the area with the greatest amplitude for the formation of the ME. Various proportions between the amounts of Labrasol^®^ and Transcutol^®^ P were considered (S_mix_ of 1:1, 2:1, 3:1, 1:2) (Figure 3). Results revealed that the maximum area for the formation of an ME occurs with a surfactant and co-surfactant ratio of 2:1; therefore, it was consequently selected as S_mix_ and optimized for the preparation of APR-ME.

The final composition of APR-ME (Table 1) was obtained by integrating APR in Plurol^®^ oleique CC497 (6%), Labrasol^®^ (29.33%), Transcutol^®^ P (14.67%) and purified water (50%). This formula was homogeneous, transparent and showed no signs of drug precipitation.

### 2.3. APR-ME Characterization

**Figure 4:**
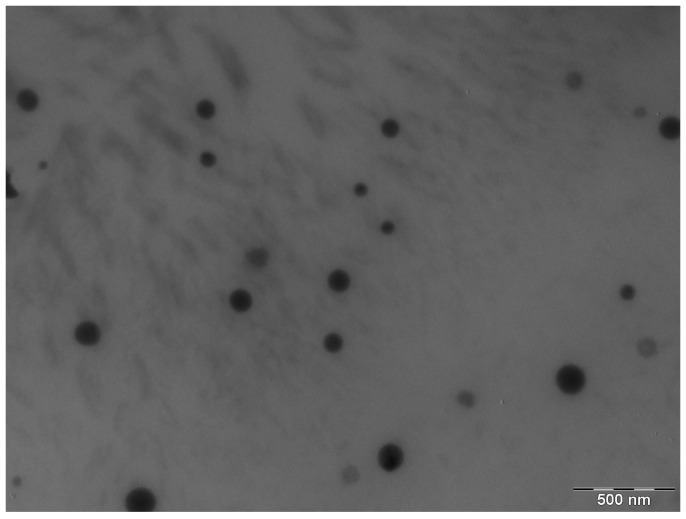
Transmission electron microscopy image of Apremilast microemulsion, magnification 40,000×.

Drug content and pH were at 99.30 ± 0.37% and 6.07 ± 0.06, respectively. After 24 h of preparation, the obtained ME was transparent with a monophasic appearance, optically isotropic and droplets with size of 60.53 ± 0.08 nm. The PDI value was 0.39 ± 0.02, which indicated the presence of a homogeneous system with droplets of uniform size. A representative TEM image of APR-ME is shown in Figure 4, in which a system of plainly distinguishable droplets of spherical shape with uniform size as consistent with DLS results can be observed. The droplets appear dark in color against a white background, with random dispersion and little agglomeration in the field.

Figure S3 shows the viscosity and rheological behavior of APR-ME. The relationship between the shear stress and the shear rate (flow curve) was linear, while the viscosity remained constant with a value of 21.58 ± 0.05 mPas·s. The mathematical model that best describes the experimental data was the Newtonian model with an *r*^2^ = 1.

### 2.4. Stability Studies

After manufacturing, MEs were stored at different temperatures (4 ± 1 °C, 30 ± 2 °C, and 40 ± 2 °C) for 90 days. The physical stability studies carried out using TurbiScanLab^®^ equipment did not show evidence of destabilization processes such as flocculation, sedimentation or coalescence. Variations greater than 10% in the T signals would indicate destabilization processes; however, as shown in Figure S4, T signals remained constant under the studied conditions, thereby confirming physical stability of the ME system. The peaks that appear on the left and right sides of the graph are formed by the contact between the sample and the glass. As such, the left region of the graph represents the bottom of the vial and the right region corresponds to the top of the vial [31,32].

The chemical stability evaluation (Table 2) showed that the drug content of APR-ME remained stable during the time of the stability study, especially at 4 °C, while at 30 °C and 40 °C there was an insignificant decrease in drug of around 1% and 1.5%, respectively.

### 2.5. In Vitro Release Studies

**Figure 5:**
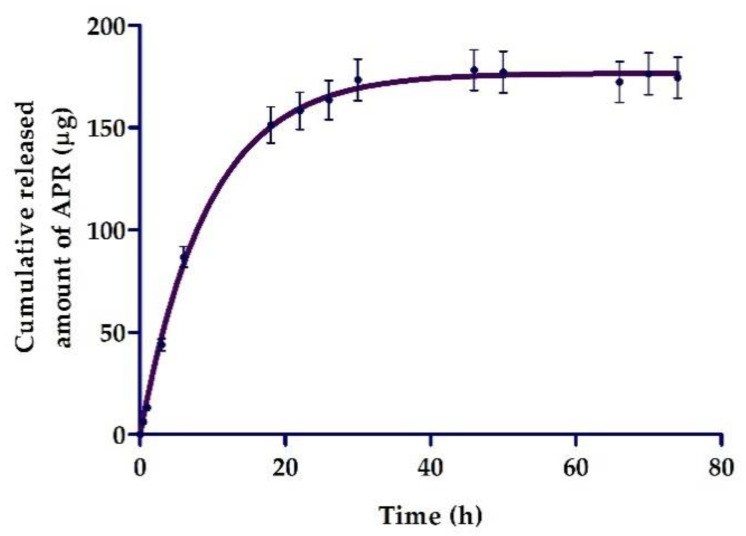
In vitro release profile of apremilast (APR) from microemulsion.

Figure 5 shows the patterns of release of APR from the ME. The graphical representation of cumulative released amount of APR vs. time indicated a faster diffusion of the drug during the first 20 h followed by a sustained release of the drug, showing a release of 174.32 µg after 74 h, which represents 58% of the drug placed in the donor compartment. The mathematical fitting suggested a first-order kinetic model (Fickian kinetic), with *r*^2^ = 0.97, a maximum release amount (Y_max_) = 176.5 µg and a release constant (K) = 0.1061 h^−1^.

### 2.6. Permeation and Q_ret_ Studies in Ex Vivo Human Skin

Apremilast was not detected (LOD: 1.13 ± 1.04 µg/mL) in the receptor fluid after 24 h of permeation test. However, it was possible to observe its presence into the skin after its extraction. The retained amount drug in the skin (Q_ret_) was 479.35 ± 102.85 µg APR/g skin/cm^2^.

### 2.7. In Vitro Anti-Inflammatory Efficacy Studies

**Figure 6:**
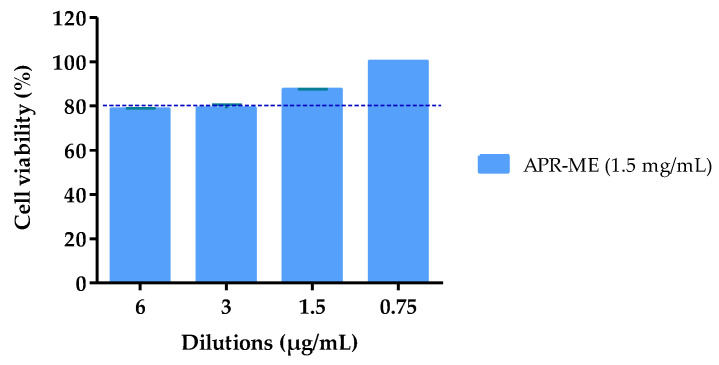
Percentage of cell viability of HaCaT cell line exposed to APR-ME for 24 h at different concentrations ranging from 6 to 0.75 µg/mL. Results obtained with Blank-ME indicated 100% viability.

**Figure 7:**
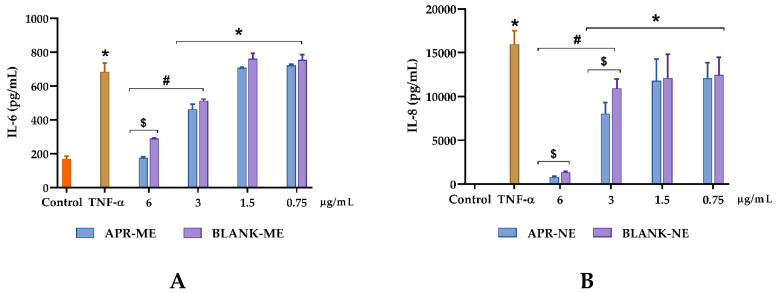
Inhibition of inflammatory response of cytokines in HaCaT cell line. (**A**) Interleukin-6 (IL-6); and (**B**) Interleukin 8 (IL-8). *Control −:* untreated cells; *Control +:* positive control. *APR-ME:* cells treated with apremilast microemulsion; and *Blank-ME:* cells treated with drug free vehicle. Results are expressed as mean ± SD (*n* = 3) Statistically significant difference: *, comparison with negative control; #, comparison between concentrations; and $, comparison between APR-ME and Blank-ME.

In order to evaluate the capability of APR-ME to inhibit the inflammatory response, IL-8 and IL-6 cytokines were measured in supernatants of TNF- α-stimulated HaCaT cells. A cell viability greater than 80% was observed in the dilutions made of APR-ME (Figure 6). In absence of APR-ME (positive control), TNF-α induced a significant increase in both analyzed cytokines. However, a decrease in IL-8 and IL-6 production dependent on drug concentration was observed in HaCaT cells stimulated with different concentrations of APR-ME (Figure 7). Secreted levels of IL-6 were significantly reduced using APR-ME at 6 µg/mL while IL-8 was significantly reduced using 6 µg/mL and 3 µg/mL of APR-ME. Finally, 1.75 and 0.75 µg/mL concentrations showed negligible effects.

### 2.8. In Vivo Anti-Inflammatory Efficacy Studies: Arachidonic Acid (Aa)-Induced Inflammation

**Figure 8:**
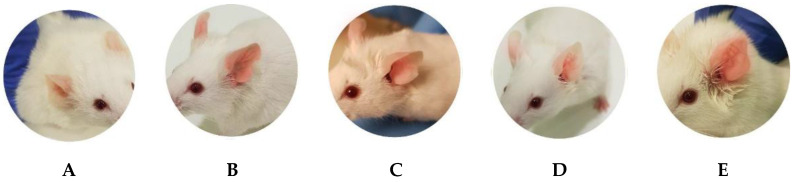
Macroscopic features of the ear’s appearance. (**A**) Negative control; (**B**) Positive control (edema and redness); (**C**) Ibuprofen group; (**D**) Apremilast microemulsion (APR-ME) group; and (**E**) Blank-ME group.

**Figure 9:**
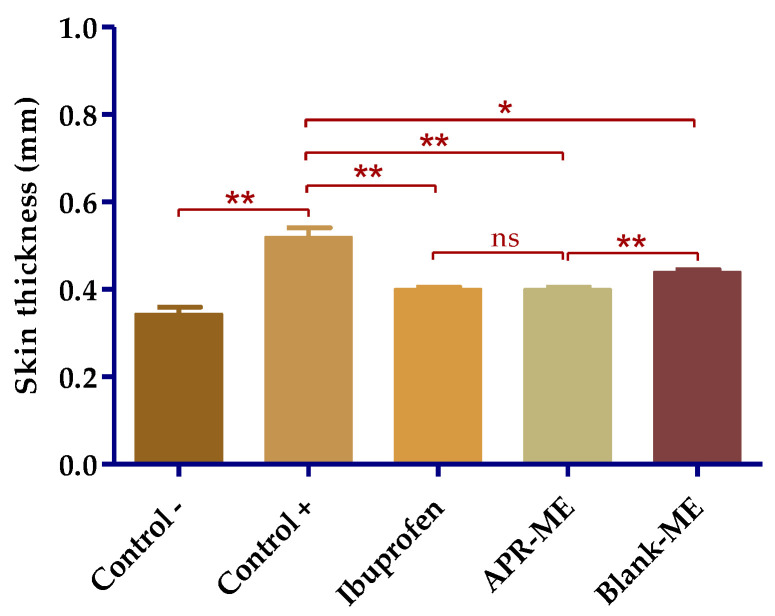
Ear thickness. Results are expressed as mean ± SD (*n* = 3). Statistically significant differences: *, *p* < 0.05; **, *p* < 0.01; ns, not significant.

The evaluation of the anti-inflammatory potential of APR-ME was performed using a model of induction of inflammation in mice ear with AA. Figure 8 shows the inflammatory action of AA, which is manifested by redness and edema in the ears of the positive control group and is substantially more evident when contrasted with those of the negative control group. Figure 9 shows noticeably greater skin thickness in the positive control group compared to the group treated with APR-ME. These results suggest that APR-ME reduces the signs of inflammation induced by the action of AA.

#### 2.8.1. Biomechanical Skin Properties Evaluation

**Figure 10:**
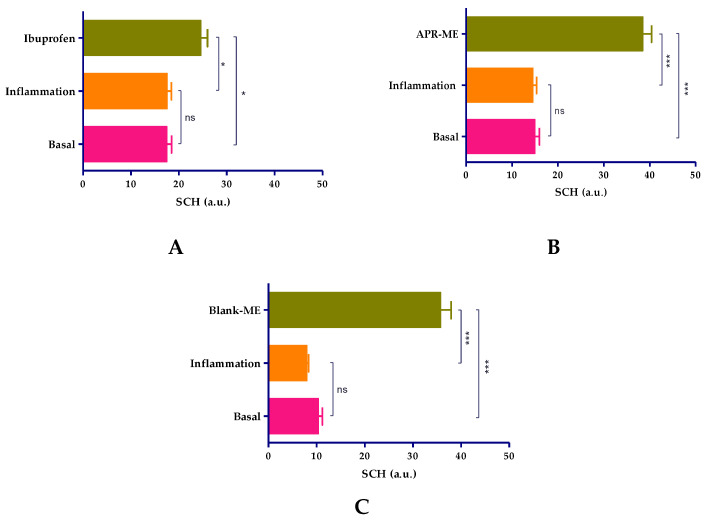
Evaluation of stratum corneum hydration (SCH). (**A**) Ibuprofen treatment group; (**B**) Apremilast microemulsion (APR-ME) treatment group; and (**C**) Blank-ME treatment group. Results are expressed as mean ± SD (*n* = 3). Statistically significant differences: *, *p* < 0.05; ***, *p* < 0.001; ns, not significant.

After inducing inflammation, stratum corneum hydration (SCH) was not significantly affected across all study groups. However, it is necessary to highlight the evident moisturizing action of different treatments, especially to APR-ME (Figure 10).

#### 2.8.2. Histological Analysis

**Figure 11:**
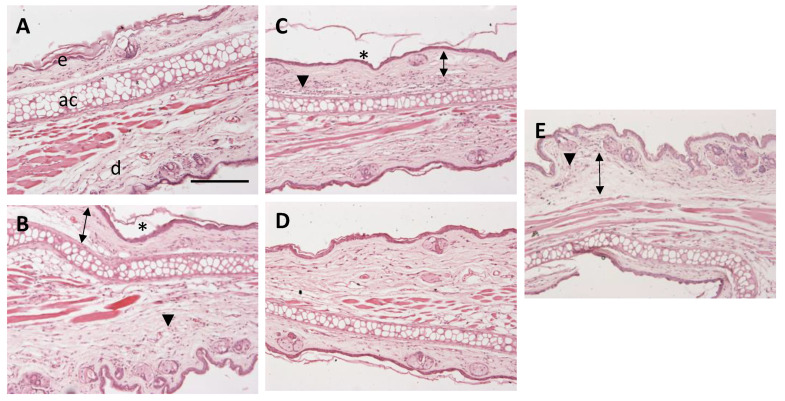
Representative micrographs of mice ear (100× magnification). (**A**) Negative control; (**B**) Positive control; (**C**) Ibuprofen; (**D**) Apremilast microemulsion (APR–ME); and (**E**) Blank–ME. Skin structures: (**e**) epidermis; (**d**) dermis; and (**ac**) auricular cartilage. Arrowheads indicate leukocytic infiltrates, arrows indicate edema, and asterisks (*) indicate loss of stratum corneum. Scale bar = 200 µM.

To examine the anti-inflammatory effect of APR-ME, hematoxylin and eosin staining histologically analyzed the architecture of mouse ear skin. Negative control micrographs consisted of a relatively thin epidermis with a contiguous stratum corneum (SC) and dermis (Figure 11A). Images of positive control exhibited edema as well as presence of leukocytic infiltrate and a slight loss of SC (Figure 11B). Loss of the SC was also evident in ibuprofen-treated mice, along with a leukocyte infiltrate (Figure 11C). When ears were topically treated with APR-ME, a profile with less inflammatory cell infiltrates and a normal SC resembling the negative control was observed (Figure 11D). Topical application of blank-NE did not improve the inflammatory characteristics induced by AA such as edema and leukocytic infiltrate (Figure 11E).

#### 2.8.3. Pro-Inflammatory Cytokines Determination

**Figure 12:**
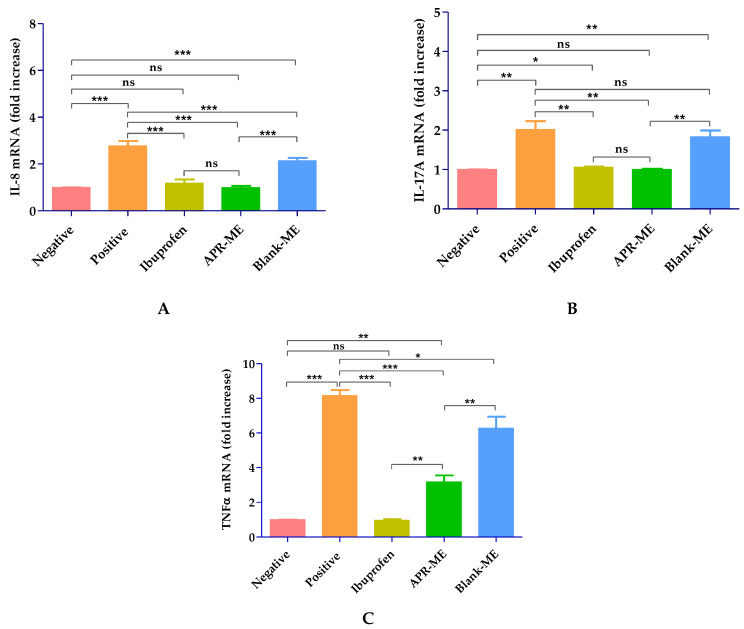
Relative expression of pro-inflammatory cytokines measured by RT-qPCR. (**A**) Interleukin-8 (IL-8); (**B**) Interleukin 17A (IL-17A); and (**C**) Tumor Necrosis Factor alpha (TNFα). *Negative*: untreated mice; *Positive*: AA-treated mice; *Ibuprofen*: mice treated with a reference anti-inflammatory product (ibuprofen gel); *Blank-ME:* mice treated with drug free vehicle; and *APR-ME:* mice treated with apremilast microemulsion. Results are expressed as mean ± SD (*n* = 4). Statistically significant difference: *, *p* < 0.05; **, *p* < 0.01; ***, *p* < 0.001; ns, not significant.

When compared with the negative control, the positive control shows a significant increase in the expression of the pro-inflammatory cytokines IL-8, IL-17A and TNFα as a result of the inflammatory process induced by topical application of AA on mouse ear. The treatment with APR-ME showed anti-inflammatory potential and was evidenced by a decrease in the production of pro-inflammatory interleukins. The anti-inflammatory efficacy between APR-ME and reference anti-inflammatory product (ibuprofen gel) did not show significant differences. Blank-ME did not demonstrate anti-inflammatory capacity; therefore, possible therapeutic action by the excipients of the formulation was discarded (Figure 12).

#### 2.8.4. In Vivo Tolerance

**Figure 13:**
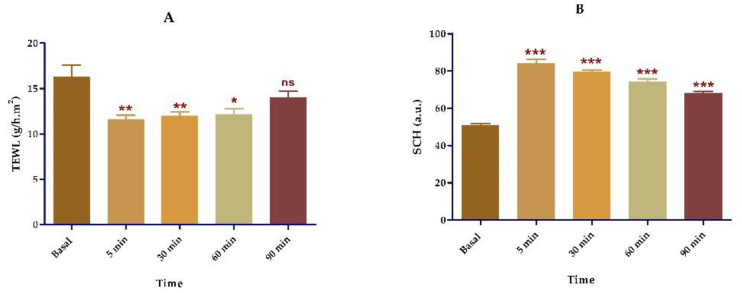
Tolerance studies in human individuals. (**A**) TEWL: Transepidermal water loss; (**B**) SCH: Stratum corneum hydration. Results are expressed as mean ± SD (*n* = 12). Statistically significant differences in comparison with basal state: *, *p* < 0.05; **, *p* < 0.01; ***, *p* < 0.001; ns, not significant.

Topical application of APR-ME on the skin of the volunteers demonstrated that the formulation does not cause signs of damage or irritation. Results showed a significant reduction in TEWL and an inversely proportional increase in SCH after APR-ME application, the latter indicating that the fatty components of ME are absorbed into the skin. These studies confirmed that the ME composition does not damage the skin barrier and can be easily absorbed into it (Figure 13).

## 3. Discussion

Inflammatory skin diseases affect numerous patients worldwide [33] not only physiologically but also psychologically and socioeconomically [34,35]. APR is an FDA approved oral drug for the treatment of adult patients with active psoriatic arthritis, adult patients with moderate-to-severe plaque psoriasis who are candidates for phototherapy or systemic therapy, and adult patients with oral ulcers associated with Behçet’s disease [36,37]. Topical application of APR as an alternative treatment for local skin inflammation was evaluated in this study. Validation of the analytical method was performed in order to obtain a reliable method for drug quantification. The results showed compliance with ICH guidelines, exhibiting good linearity in a range from 1.25 to 200 µg/mL (*r*^2^ = 0.999) in addition to having acceptable precision, accuracy and robustness [38]. Detailed results are shown in the Supplementary Materials. APR is categorized as a class IV drug, according to the Biopharmaceutical Classification System (BCS), due to its low solubility and low permeability [39]. Hence, this study incorporated APR in a ME as a strategy to improve the solubility and permeability of the drug. The formulation was developed using the excipients that showed the highest solubilizing capacity for the drug: Plurol^®^ oleique CC497, Labrasol^®^ and Transcutol^®^ P (Figure 2). The search for the most suitable components with regards to the solubility of the drug is a critical step in the development of a ME in order to ensure its stability and high drug loading capacity [40,41]. Labrasol^®^ is a non-ionic oil/water surfactant characterized by its low irritability for the skin and which is commonly used with Plurol^®^ oleique in dermal formulations [42]. Transcutol^®^ P was selected as a co-surfactant due to its ability to solubilize APR with non-toxic and biocompatible properties with the skin [43]. The pseudo-ternary phase diagram with a surfactant-cosurfactant mixture (S_mix_) in the ratio 2:1 (*w*:*w*) showed a greater ME region (Figure 3), and therefore was used for drug incorporation. APR-ME (1.5 mg/mL) was prepared by the titration method, where there is a spontaneous emulsification by a low energy process [44]. Final formulation was homogeneous and transparent, with a slightly acidic pH, which is biocompatible with the skin and thus suggests that APR-ME does not have an irritating effect [45]. The physical characterization by DLS and TEM (Figure 4) showed the presence of droplets of nanometric size and spherical shape distributed uniformly. The microemulsion presented isotropic characteristics [46] after its evaluation with a polarized light microscope. The rheological behavior of dermal formulations influences administration properties such as sensory characteristics, spreadness and dosage [47,48]. In ME systems, evaluation of rheological properties provides information on the structural constitution as well as the interactions between droplets [49,50]. The rheological behavior of APR-ME (Figure S3) was determined from the relationship between the shear stress and the shear rate (flow curve), which was linear, while the viscosity remained constant. This result was supported by mathematical modeling which confirmed Newtonian behavior that is typical for this type of formulation and thus providing the possibility of being easily administered by spray.

The transmission profiles obtained by multiple light scattering analysis allowed assessment and demonstration of the physical stability of APR-ME for a period of 90 days due to the fact that no signals of creaming, sedimentation, flocculation or coalescence were detected (Figure S4). Additional evaluation of physical parameters including droplet size, PDI and pH confirmed the high physical stability of the formulation. APR-ME also showed chemical stability, since there were insubstantial changes in the quantified drug content after 90 days at different temperatures, which demonstrates the high compatibility between the components with the drug.

In dermal formulations, release and permeation studies of drug are useful tools to predict drug bioavailability while consequently determining its efficacy [51,52]. In this work, APR was released from NE following a first-order model (Fickian kinetic) in which the release rate is directly proportional to the concentration of the remaining drug (Figure 5). As such, compliance with Fick’s law is observed, which establishes that the diffusion rate through a membrane is directly proportional to the concentration gradient of the substance on both sides [53]. This result confirmed that the formulation can release the incorporated drug and thus it does not limit drug permeation through the skin.

Ex vivo permeation studies using human skin and under suitable conditions are successfully used to predict in vivo behavior of topical formulations [54,55]. The results of this assay revealed that APR-ME could be used as an effective local treatment for skin inflammation since the drug was able to cross the SC and remain retained into the skin (479.35 ± 102.85 µg APR/g skin/cm^2^) without reaching the receptor compartment. These findings suggest that it is possible to reach adequate drug concentration in the target area while avoiding systemic adverse effects.

Regarding the therapeutic efficacy, both in vitro and in vivo tests APR-ME demonstrated anti-inflammatory potential after its application (Figure 7 and Figure 12). In vitro efficacy studies using HaCaT cell line corroborated the activity of the drug on the expression of previously reported pro-inflammatory interleukins such as IL-6 and IL-8 [56]. The inhibitory effects of APR-ME on the production of inflammatory mediators were accompanied by concentration-dependent decreases in IL-6 and IL-8 mRNA and protein expression levels. Additionally, in vivo efficacy studies were performed using AA to induce inflammation in the mice ear. AA is a long-chain unsaturated essential fatty acid that is formed from the synthesis of linoleic acid in the diet, and is considered one of the precursors of prostaglandins, thromboxanes and leukotrienes [57,58]. Research supports the use of AA for the induction of inflammatory processes because it is an intrinsic component of the inflammatory response, which can subsequently be used in mouse ear edema models for evaluation of anti-inflammatory potential of drugs [59,60,61,62]. APR, being a phosphodiesterase 4 inhibitor, elevates intracellular cAMP levels, which in turn intervenes in the regulation of the inflammatory response by modulating the expression of TNFα and IL-17 [63]. The skin inflammatory process promotes skin dryness, induces infiltration of inflammatory cells, increases skin thickening and stimulates the membrane receptors of keratinocytes which promotes the release of various inflammatory mediators such as TNFα, IL-8, IL-1α, IL-6 and IL-12 [64,65]. In our study, SCH results (Figure 10) showed that the SC water content was not altered for the topical application of AA; however, a moisturizing effect was noticeable in mice treated with APR-ME. Nevertheless, AA induced an increase in the skin thickness, which is indicative of edema, hyperplasia and intensified vascular permeability (Figure 8) [66]. This effect was corroborated by histological studies (Figure 11) that showed the presence of edema and leukocytic infiltrate in the positive control. The therapeutic efficacy of the formulation in the treatment of these symptoms was confirmed in this study, since a reduction of skin thickness (Figure 9) as well as in the infiltration of inflammatory cells was observed after topical administration of APR-ME. Regarding the immunological mechanisms involved in the local inflammatory process induced by AA, an increase in the expression of pro-inflammatory cytokines IL-8, IL-17A and TNFα compared to the negative control group was observed (Figure 12). Topical treatment with APR-ME on the inflamed area significantly reduced the expression of these cytokines. The role of IL-8, IL-17A and TNFα in skin inflammation has been widely described in previous studies [67,68,69]. TNF-α is a multifunctional agent that stimulates the acute phase of an inflammatory reaction and is secreted by different cell types, including monocytes, macrophages, Langerhans cells and microglia [70,71]. This cytokine is one of the most abundant early markers and is considered a “master regulator” in inflammatory processes because it can trigger local expressions of other pro-inflammatory cytokines such as IL-1, IL-6 and IL-8 [72,73,74]. IL-8 is an inflammatory chemokine that is produced by various cells, including monocytes, fibroblasts, endothelial cells, keratinocytes and chondrocytes [75]. The role of IL-8 in inflammation and inflammatory skin diseases has been confirmed by experimental models that showed a constitutive expression of IL-8 mRNA in normal cultured keratinocytes along with a rapid increase in its level when submitting a stimulus, such as irradiation [76]. Finally, IL-17 plays an important role in the regulation of innate and adaptive immune systems. However, its overexpression is involved in various inflammatory and autoimmune diseases including dermatitis and psoriasis [77,78,79]. In summary, the efficacy studies carried out in this work confirmed: (i) the therapeutic effect of APR in an in vitro model in HaCaT cell lines; (ii) no apparent cytotoxicity (cell viability [80,81,82] Figure 6) of APR-ME in concentrations from 6 µg/mL onwards; (iii) the therapeutic potential of APR-ME that was evidenced by a decrease in the expression of the cytokines IL-8, IL-17A and TNFα; reduction in the infiltration of inflammatory cells as well as in edema and redness; and (iv) moisturizing effect on affected area thanks to the composition of the formulation based on oils and surfactants.

Finally, the tolerance in vivo study (Figure 13) was conducted, where biochemical skin properties were analyzed in healthy volunteers. TEWL and SCH have been used in dermatology to detect possible irritant effects of topical formulations [83,84,85]. Normal hydration levels maintain SC flexibility and its viscoelastic characteristics in addition to facilitating the enzymatic reactions involved in the maturation of corneocytes [86,87]. Destabilization or damage to the skin surface by exposure to physical or chemical agents can cause changes in TEWL and SCH [88,89,90]. In this study, the evaluation of these parameters confirmed that APR-ME does not alter sebaceous function nor compromise the integrity of the skin barrier. On the contrary, improved hydration levels were evidenced by a significant reduction of TEWL along with an increase of SCH following topical application of APR-ME, likely due to the moisturizing effect of the formulation.

## 4. Materials and Methods

### 4.1. Materials

Apremilast (purity of 99.6% and molecular weight of 460.5 Da) was purchased from Wuhan Senwayer Century Chemical (Wuhan, China). Polyglyceryl-3 dioleate (Plurol^®^ oleique CC497), oleoyl polyoxyl-6 glycerides (Labrafil^®^ M1944 CS), propylene glycol monolaurate-type II (Lauroglycol™ 90), caprylocaproyl polyoxyl-8 glycerides (Labrasol^®^) and diethylene glycol monoethyl ether (Transcutol^®^ P) were supplied by Gattefossé (Saint-Priest, France). Castor oil (*Ricinus communis* L.), polyethylene glycol sorbitan monooleate (Tween^®^ 80) and propylene glycol 400 were supplied by Sigma-Aldrich (Madrid, Spain). Macrogol glycerol ricinoleate [Cremophor^®^ EL] was supplied by Fagron Iberica (Barcelona, Spain). Reagents for histological procedures were purchased from Sigma and Thermo Fisher Scientific (Barcelona, Spain). HaCaT cell line was purchased from Cell Line Services (Eppelheim, Germany) and the reagents used for cell cultures were obtained from Gibco (Carcavelos, Portugal). Reagents for MTT assay were obtained from Invitrogen Alfagene^®^ (Carcavelos, Portugal). A Millipore Milli-Q^®^ purification system (Millipore Corporation; Burlington MA) was used to obtain ultrapure water for all experiments. Finally, the reagents used in this study were of analytical grade.

### 4.2. Validation of the Analytical Method

High-performance liquid chromatography (HPLC) method for APR quantification was validated. The HPLC system consisted of a Waters 1525 pump and a UV-VIS 2487 detector (Waters, Milford, MA, USA). Data was collected and processed using Empower Pro software (Waters, Milford, MA, USA). Analysis was performed with a Kromasil C18 chromatographic column (250 mm, 4.6 mm and 5 µm). The mobile phase consisted of a mixture of acetonitrile (ACN) water (70:30 *v*/*v*) filtered with a 0.45 µm PVDF membrane filter (Millipore Corp., Madrid, Spain). The mobile phase was pumped at a flow rate of 1 mL/min and the injection volume was 20 µL. The isocratic elution was carried out at 25 °C and the detection was carried out by UV spectrophotometry at ʎ = 230 nm.

#### Conditions Analyzed

Standard stock solution (APR = 250 µg/mL) was prepared daily in ACN. The calibration curve was prepared using mobile phase to obtain dilutions in a concentration range of 1.25 to 200 µg/mL. The method was validated in terms of linearity, precision, accuracy, robustness, sensitivity and specificity. Validation was performed according to the International Conference on Harmonization (ICH) Q2A and Q2B guidelines.

*Linearity:* The linearity was evaluated by a one-way ANOVA test to compare peak areas versus nominal concentrations of each standard [91]. Differences were considered statistically significant when *p* ˂ 0.05. The least-square linear regression analysis and mathematical determinations were carried out using GraphPad Prism v5.0 software (GraphPad Software Inc., San Diego, CA, USA).

*Accuracy and Precision:* Accuracy and precision were investigated by measuring samples in the concentrations of 1.25 to 200 μg/mL. The between-day test for three days was performed to analyze the analyte within all the previously mentioned concentrations. Precision was defined as the relative standard deviation (RSD%) of the measurement, whereas accuracy was expressed as a relative error (RE%), using the equation:

(1) $$RE\%=\frac{X_{a}-X_{r}}{X_{a}}\cdot 100$$

where: $X_{a}$ is the theoretical value of the concentration, $X_{r}$ is the experimental value of the concentration and 100 is the constant.

*Robustness:* Robustness was determined by changing the experimental conditions of concentration of the mobile phase by varying the proportions by ±1% of acetonitrile and water. The effects of these variations in the experimental conditions were tested for the retention time. SD was calculated.

*Specificity:* The specificity of the method was evaluated by analyzing any possible interferences due to the components of the skin that are released during the passage of the drug. Different samples were evaluated including drug-free mobile phase and standard 100 µg/mL, as well as two samples obtained from permeation studies: APR retained in the skin and a skin blank (sample extracted from receptor compartment in a Franz cell using drug-free skin) as a control. A volume of 20 µL of each sample was injected and then the chromatographic profiles (ʎ = 230 nm) were analyzed.

*Sensitivity:* Sensitivity was analyzed using limit of detection (LOD) and limit of quantification (LOQ). The LOD and LOQ were calculated based on each of the standard deviation of the response and the slope of the calibration curve. LOD and LOQ are calculated using:

(2) $$LOD\;or\;LOQ=k\frac{SD_{Sa}}{S_{b}}$$

where: k is the factor related to the level of confidence (*k* = 3.3 and 10 for LOD and LOQ, respectively). $SD_{Sa}$ is standard deviation of the intercept. $S_{b}$ is the slope.

### 4.3. Solubility Studies

The solubility of APR in various oils (Plurol^®^ oleique CC497, Labrafil^®^ M1944 CS, Lauroglycol™ 90 and Castor oil), surfactants (Tween^®^ 80, Labrasol^®^ and Cremophor^®^ EL) and co-surfactants (Transcutol^®^ P and Propylene glycol 400) was determined. An excess of APR was added to the indicated vehicles and mixed under stirring for 4 h. The samples were equilibrated for 24 h and later centrifuged at 9000 rpm for 10 min. The supernatant was diluted with methanol to quantify the amount of APR dissolved using a Thermo Spectronic Helios Beta UV-Visible Spectrophotometer (Thermo Fisher Scientific, Karlsruhe, Germany).

### 4.4. Pseudo-Ternary Phase Diagrams

Four pseudo-ternary phase diagrams were constructed with the components that presented higher APR solubility capacity by the water titration method [92]. The pseudo-ternary diagrams were constructed using Plurol^®^ oleique CC497 as the oil phase, a mixture of Labrasol^®^ and Transcutol^®^ P (S_mix_) as surfactants and co-surfactants, respectively, and purified water as the aqueous phase. Labrasol and Transcutol^®^ P were mixed at ratios 1:1, 2:1, 3:1 and 1:2 while oil and S_mix_ were mixed at a fixed weight ratio 9:1, 8:2, 7:3, 6:4, 5:5, 4:6, 3:7, 2:8 and 1:9 (*w*/*w*); Titration with water was carried until turbidity or phase separation was observed in each oil/S_mix_ ratio. The single-phase and transparent combinations were placed within the emulsification area, which made it possible to delimit the ME formation region. The diagram showing the highest emulsification area was selected as S_mix_ optimized.

### 4.5. Preparation of Apremilast Microemulsion (APR-ME)

APR-ME (1.5 mg/mL) was prepared by incorporating the APR into the oil and S_mix_ under stirring at 700 rpm until drug dissolution. Afterwards, purified water was added dropwise under constant stirring until a transparent ME was obtained.

### 4.6. Characterization of the Apremilast Microemulsion

#### 4.6.1. Content and pH of APR-ME

The drug content in the ME was quantified using the HPLC method described in Section 4.2. The pH values of the formulation were determined using a calibrated digital pH meter GLP 22 (Crison Instruments, Barcelona, Spain).

#### 4.6.2. Drop Size and Polydispersity Index

The droplet size and polydispersity index (PDI) were determined using 1 mL of APR-ME without dilution at 25 °C by dynamic light scattering technique (DLS) with a Zetasizer Nano ZS kit (Malvern Instruments, Malvern, UK). Previously, the formulation refractive index was measured obtaining a value of 1.397. Values are reported as the mean ± SD of three replicates.

#### 4.6.3. Transmission Electron Microscopy (TEM)

To corroborate droplet size and to examine morphology, the formulation was analyzed by TEM using a JEOL JEM-1010 microscope (JEOL Ltd., Tokyo, Japan). For negative staining, a drop of undiluted ME was deposited on a Formvar coated copper grid for 1 min. After blotting the excess of formulation, the grid was washed with a drop of ultrapure water. Then, the sample was stained with UranyLess^®^ for 1 min. Finally, TEM images were taken after 24 of drying.

#### 4.6.4. Viscosity and Rheological Behavior

Viscosity and rheological behavior were analyzed without dilution at 25 °C using a Haake RheoStress 1 rheometer (Thermo Fisher Scientific, Karlsruhe, Germany). ME was assessed in duplicate using a program consisting of three steps: an increasing period of shear rate (0–50 s^−1^) for 3 min, followed by a period of constant shear rate at 50 s^−1^ for 1 min, and a period of decreasing shear rate (50–0 s^−1^) for 3 min. Values are reported as the mean ± SD of three replicates. Results of the flow curves were fitted to various mathematical models:

Newton Equation:

(3) $$\tau =\eta \cdot \dot{\gamma }$$

Bingham Equation:

(4) $$\tau =\eta \cdot \dot{\gamma }$$

(5) $$\tau =\tau_{0}+(\eta_{p}\cdot \dot{\gamma )}$$

Ostwald-de-Waele Equation:

(6) $$\tau =K\cdot {\dot{\gamma }}^{n}$$

Herschel-Bulkley Equation:

(7) $$\tau =\tau_{0}+K\cdot {\dot{\gamma }}^{n}$$

Casson Equation:

(8) $$\tau =\sqrt[{n}]{\left(\tau_{0}^{n}+{\left(\eta_{0}\cdot \dot{\gamma }\right)}^{n}\right)}$$

Cross Equation:

(9) $$\tau =\dot{\gamma }\cdot (\eta_{\infty }+(\eta_{0}-\eta_{\infty })/(1+{\left(\dot{\gamma }/{\dot{\gamma }}_{0}\right)}^{n})$$

where: τ is the shear stress (Pa). $\dot{\gamma }$ is the shear rate (1/s). η is the dynamic viscosity (mPa∙s). $\tau_{0}$ is the yield shear stress (Pa). $\eta_{0}$ is the zero-shear rate viscosity. $\eta_{p}$ is a constant plastic viscosity (mPa∙s). $\eta_{\infty }$ is the infinity shear rate viscosity. η is the flow index. *K* is the consistency index.

### 4.7. Stability Study

The physical stability of APR-ME was evaluated by analyzing transmission profiles (T) obtained by multiple light scattering using the TurbiScanLab^®^ equipment (Formulation, L’Union, France), whose light source is near-infrared (λ = 880 nm). The TurbiScanLab^®^ is an optical instrument that characterizes emulsions and dispersions which is based on the measurement of backscatter (BS) and transmission (T) signals in order to early detect destabilization phenomena such as droplet aggregation or their migration [93]. The T profiles of the APR-ME samples (20 mL) stored at 4, 30 and 40 °C were measured at predetermined time intervals (1, 30, 60 and 90 days).

The chemical stability of APR-ME was evaluated by the quantification of the drug present in the formula at all the indicated time intervals and storage temperatures. To that end, 1 mL of ME was taken and diluted with ACN at a ratio of 1:100. This sample was analyzed according to the method described in Section 4.2.

### 4.8. In Vitro Release Study

The release study of APR from ME was performed using Franz vertical diffusion cells of 13 mL capacity (Franz Diffusion Cells 400; Crown Glass, Somerville, NJ) and dialysis membranes (MWCO 12 KDa) previously hydrated for 24 h with methanol/water (50:50 *v*/*v*), which were washed with water and mounted between the donor and receptor compartment. The effective diffusion area was 2.54 cm^2^. The receptor compartment was filled with Transcutol^®^ P/water medium (60:40 *v*/*v*), which was kept under continuous stirring (700 r.p.m.). 0.2 mL samples of APR-ME were placed in the donor compartment and the system was maintained at 32 ± 0.5 °C, to simulate skin conditions in vivo. Aliquots of 0.2 mL were withdrawn from the receptor compartment at predetermined time intervals up to 74 h, which were subsequently replaced by the same volume of receptor medium. The concentration of released APR from ME was quantified by HPLC (Section 4.2). Values are reported as the mean ± SD of three replicates. The data were fitted to various mathematical models to determine the release kinetics, where the model was selected based on the correlation coefficient (*r*^2^):

Zero Order Equation:

(10) $$Q_{t}=K_{0}\cdot t+Q_{\infty }$$

First Order Equation:

(11) $$Q_{t}=Q_{\infty }\left(1-e^{-K_{f}\cdot t}\right)$$

Higuchi’s Equation:

(12) $$Q_{t}=K_{H}\;t^{\frac{1}{2}}$$

Korsmeyer–Peppas Equation:

(13) $$Q_{t}=K_{k}\;t^{n}$$

where: $Q_{t}$ is the amount of released drug at time t. $Q_{\infty }$ is the maximum amount of release drug. $K_{0},K_{f},K_{H},K_{k}$ are the constants of release rate. t is time in hours. n is the exponent of release (related to the drug release mechanism) n≤0.43 (Fickian diffusion), 0.43 < n < 0.85 (abnormal transport) and ≥ 0.85 (case II transport; zero-order release kinetic). $r^{2}$ is the determination coefficient.

### 4.9. Ex Vivo Skin Permeation Study

This assay was performed using human skin from an abdominal lipectomy of a healthy 38-year-old woman (Hospital de Barcelona, SCIAS, Barcelona, Spain) under prior written consent in accordance with the Ethics Committee of the Hospital de Barcelona (number 001, dated January 20, 2016). The integrity of the skin samples was evaluated based on transepidermal water loss (TEWL) using Tewameter TM 300 (Courage & Khazaka Electronics GmbH, Cologne, Germany) and those with results less than 10 g/m^2^/h were used [94]. For the experiment, the skin samples were cut with the help of an Aesculap GA 630 dermatome (Aesculap, Tuttlingen, Germany) in order to obtain skin samples 0.4 mm thick, which were then placed between the donor and receptor compartment of Franz diffusion cells (6 mL) with a diffusion area of 0.64 cm^2^. The receptor medium consisted of a solution of Transcutol^®^ P/water (60:40, *v*/*v*) at 32 ± 0.5 °C and stirring at 700 r.p.m. to guarantee sink conditions. 0.2 mL samples of APR-ME (1.5 mg/mL) were placed in the donor compartment in contact with the outer surface of the skin. Aliquots of 0.2 mL were withdrawn from the receptor compartment at predetermined time intervals up to 30 h, which were then replaced by the same volume of receptor medium. The amount of permeated APR was quantified by HPLC (Section 4.2). Values are reported as the mean ± SD of three replicates.

#### Determination of the Amount of Drug Retained in the Skin

At the end of the permeation study, the amount of APR retained in the skin (Q_ret_ µg/g skin/cm^2^) was extracted by ultrasound-assisted extraction. The skin samples were removed from Franz diffusion cells and cleaned with gauze soaked in a 0.05% solution of dodecyl sulfate and washed with distilled water. The skin permeation area was cut, weighed and immersed in 1 mL of ACN for 30 min using an ultrasonic bath. Solvent samples were filtered and quantified by HPLC (described in Section 4.2).

### 4.10. In Vitro Anti-Inflammatory Efficacy Studies in HaCaT Cell Line

#### 4.10.1. Cell Culture

All assays were performed with immortalized keratinocytes HaCaT cell line. The cells were grown in Dubelcco’s Modified Eagle’s Medium (DMEM) with high glucose content buffered with 25 mM HEPES, and supplement with 1% non-essential amino acids, 100 U/mL penicillin, 100 g/mL streptomycin and 10% heat inactivated Fetal Bovine Serum (FBS). The cells were growth until 80–90% confluence at 37 °C under 5% CO_2_ atmosphere. The culture medium was changed every 3 days.

#### 4.10.2. Cell Viability Assay

The effect of APR-ME on cell viability was evaluated using an Methylthiazolyldiphenyl-tetrazolium bromide assay (MTT assay). HaCaT cell line (2 × 10^5^ cells/mL) were seeded in 96-well plates (Corning) while being kept in a humidified incubator at 37 °C under a 5% CO_2_ atmosphere for 48 h to allow adhesion. Experiments were performed at 80%–90% confluence.

APR-ME (1500 µg/mL) was diluted to obtain a concentration range of 6 to 0.75 µg/mL in order to select the concentrations that guarantee a cell viability greater than 80% for posterior in vitro anti-inflammatory studies. After 24 h of incubation with these dilutions, the HaCaT cells were washed with 1% sterile PBS and incubated with MTT (Sigma-Aldrich Chemical Co, St. Louis, MO, USA) solution (5 mg/mL) for 2 h at 37 °C. Afterwards, the medium was carefully removed and 0.1 mL of Dimethyl sulfoxide (DMSO, purity 99%) was add to lyse the cells and dissolve the purple MTT crystals. Cell viability was measured at 570 nm in a microplate photometer Varioskan™ LUX (Thermo Scientific, Waltham, MS, USA). In parallel, a negative control (cells without any stimulation or treatment) was processed for comparison. The results were expressed as percentage of cell survival relative to the control (untreated HaCaT cells, 100% viability) using the following equation:

(14) $$\mathrm{Cell}\;\mathrm{viability}=\left[\frac{\mathrm{ABS}\;\mathrm{treated}\;\mathrm{cells}}{\mathrm{ABS}\;\mathrm{control}\;\mathrm{cells}}\right]\times 100$$

#### 4.10.3. In Vitro Anti-Inflammatory Efficacy

In order to determine the in vitro anti-inflammatory effect of APR-ME, HaCaT cells (2 × 10^5^ cell/mL) were seeded in a 12-well plate and grown until 80%–90% confluence. Different concentrations of APR-ME were added in presence of TNF-α (50 ng/mL). Cells only stimulated with TNF- α were considered as positive control and untreated cells as the negative control. After 24 h of incubation, supernatants were collected and centrifuged (10,000× *g* for 15 min at 4 °C) and stored at −80 °C until use. Secreted levels of the pro-inflammatory cytokines IL-8 and IL-6 were measured using enzyme-linked immunosorbent assay (ELISA) sets (BD Biosciences, CA, USA), according to manufacturer’s instructions. The results were expressed as pg/mL.

### 4.11. In Vivo Anti-Inflammatory Efficacy Studies: Arachidonic Acid (AA)-Induced Inflammation

#### 4.11.1. Animals and Study Protocol

The study protocol was approved by the Animal Experimentation Ethics Committee of the University of Barcelona (CEEA/UB ref. 4/16 and Generalitat ref. 8756. Date: 28 January 2016). The experiment was performed to evaluate the efficacy of APR-ME for the treatment of skin inflammation using BALB/c mice (4–5 months old) (n = 12) divided into four cages according to experimental groups (n = 3) in a temperature and humidity-controlled room with food and water *ad libitum*. These groups included: negative control (untreated healthy animals), positive control (treatment only with AA), APR-ME group (treatment with APR-ME after inducing inflammation), Blank-ME group (treatment with the vehicle after inducing inflammation) and ibuprofen group (treatment with ibuprofen gel 50 mg/g; reference: 886192.7 after inducing inflammation). Inflammation was induced in the mice corresponding to the following groups: APR-ME, Blank-ME, Ibuprofen, and positive control, by direct application of 60 µL of AA dissolved in PBS (5 mg/mL). After 20 min, biomechanical properties were analyzed and APR-ME, Blank-ME, or ibuprofen gel was topically applied over the inflamed area. The trial concluded 20 min later with biomechanical properties, evaluation, and sacrifice by cervical dislocation of the animals. Additionally, a negative control group (healthy mice) without any treatment was used to compare the results. Extraction of the biopsy samples from mice ears was performed for histological examination and analysis of gene expression of pro-inflammatory cytokines by quantitative reverse transcription polymerase chain reaction (RT-qPCR). The thickness of the mice ear of each group was measured using a Digital Thickness Gauge of 0–10 mm (Mitutoyo, Japan).

#### 4.11.2. Biomechanical Skin Properties Evaluation

Stratum corneum hydration (SCH) was measured in basal state as well as after inducing inflammation with AA, and after treatment with APR-ME, Blank-ME, or ibuprofen gel, using a Corneometer CM-825 (Courage & Khazaka Electronics GmbH, Germany).

#### 4.11.3. Histological Analysis

The ear biopsies carefully extracted from mice were stored for 24 h in 4% formaldehyde at room temperature, suspended in PBS for 3 h (replacing it with fresh medium in time intervals of 1 h) and stored in 96% ethanol. Afterwards, the samples were embedded in paraffin blocks, cut into 6 µm sections and stained with hematoxylin and eosin. Finally, the samples were observed under a microscope to assess the structure of the skin and possible inflammatory responses using Olympus BX41 microscope equipped with Olympus XC50 camera.

#### 4.11.4. Pro-Inflammatory Cytokines Study

Total RNA was isolated from ear samples using the TRIZol^®^ method (Thermo Fisher Scientific, Waltham, MA, USA). To do so, small fragments of tissue were homogenized using 1 mL of cold TRIZol reagent and under the Polytron homogenizer for 3 min, as manufacturer instructions specified. RNA concentration and quality were tested using the NanoDropTM 2000/2000c spectrophotometer (Thermo Fisher Scientific, Waltham, MA, USA).

Total RNA (1 µg) was reverse transcribed into cDNA using the Revert Aid First Strand cDNA synthesis kit (Thermo Fisher Scientific, Waltham, MA). Subsequently, qPCR was performed using StepOnePlus Real-Time PCR (Applied Biosystem, Foster City, CA, USA) and the primers for IL8, IL-17A and TNFα (Table 3). GAPDH was used as maintenance, and the conditions of the PCR cycles included: 5 min at 94 °C for denaturation, 30 cycles of amplification at 72 °C for 2 min, 1 min at 94 °C, 1 min at 60 °C and a final cycle at 72 °C for 10 min for the final extension. Finally, the relative gene expression of each gene was normalized with housekeeping GAPDH, and the formula 2^−ΔΔCt^ was used to calculate the changes.

### 4.12. In Vivo Tolerance Study in Humans

A tolerance study was carried out in 12 volunteers (6 men and 6 women) with healthy skin between 20 and 30 years old with prior written informed consent. This study was approved by the Ethics Committee of the University of Barcelona (IRB00003099) in accordance with the recommendations of the Declaration of Helsinki. TEWL and SCH were measured on the ventral forearm area at baseline and after topical application of APR-ME at predetermined time intervals of 5, 30, 60 and 90 min. The readings were recorded using a Tewameter^®^ TM300 and Corneometer^®^ 825 (Courage & Khazaka Electronics GmbH, Cologne, Germany) for TEWL and SCH, respectively.

To measure TEWL, the probe was pressed and held on the skin for 2 min and the results are expressed as g/cm^2^/h. For the SCH value, the probe was pressed onto the skin to measure the dielectric constant of the skin in which measurements were given in arbitrary units (AU). TEWL and SCH results were recorded as the mean ± SD (*n* = 12).

### 4.13. Statistical Analysis

Statistical analysis was performed with GraphPad Prism, v5.0 software (GraphPad Software Inc., San Diego, CA, USA). The results are presented as the mean ± SD (*n* = 3). The experimental data obtained was analyzed using a one-way ANOVA followed by Tukey’s test to compare the mean values. A value of *p* less than 0.05 was established to consider statistically significant differences.

## 5. Conclusions

The results of this study support the topical use of APR-ME as an appealing therapeutic strategy in the treatment of local skin inflammation. This homogeneous and transparent formulation exhibited a Newtonian behavior, thus allowing easy administration via spray or roll-on. Moreover, high tolerability of APR-ME in healthy volunteers was exhibited due to its composition based on approved excipients for dermal formulations with high biocompatibility with the skin. APR-ME demonstrated its capacity to release the incorporated drug following a first-order kinetic model while also guaranteeing a local anti-inflammatory effect with reduced systemic adverse effects due to the high drug retention in the skin. This anti-inflammatory potential was evidenced by a reduction in the production in vitro of IL-6 and IL-8, a decrease in the infiltration of inflammatory cells, less damage to the stratum corneum and a decrease in the expression of pro-inflammatory cytokines such as TNFα, IL -8 and IL-17 from the in vivo model.

## Figures and Tables

**Table 1:** Formula of apremilast microemulsion (APR-ME).

Components	(%)
Apremilast (1.5 mg/mL)	
Plurol^®^ oleique CC497 (Polyglyceryl-3 dioleate)	6.00
Labrasol^®^ (Caprylocaproyl Polyoxyl-8 glycerides)	29.33
Transcutol^®^ P (Diethylene glycol monoethyl ether)	14.67
Water	50.00

**Table 2:** Chemical stability studies of APR-ME.

Time (days)	Drug Content (%)		
	4 ± 1 °C	30 ± 2 °C	40 ± 2 °C
1	99.66	99.67	99.67
30	99.66	99.66	99.66
60	99.61	99.12	98.90
90	99.59	98.67	98.14

**Table 3:** Primer sequences used for real-time PCR in *Mus musculus* BALB/c.

Gene	Primer Sequence (5′ to 3′)	Gene Accession Number
GAPDH	FW: AGCTTGTCATCAACGGGAAG	BC023196.2
	RV: TTTGATGTTAGTGGGGTCTCG	
IL-8	FW: GCTGTGACCCTCTCTGTGAAG	X53798.1
	RV: CAAACTCCATCTTGTTGTGTC	
IL-17A	FW: TTTTCAGCAAGGAATGTGGA	NM_010552.3
	RV: TTCATTGTGGAGGGCAGAC	
TNFα	FW: AACTAGTGGTGCCAGCCGAT	NM_013693.3
	RV: CTTCACAGAGCAATGACTCC	

GAPDH = Glyceraldehyde–3–Phosphate Dehydrogenase; IL–8 = Interleukin–8; IL–17A = Interleukin–17A; TNFα = Tumor necrosis factor alpha; FW = forward primer; RV = reverse primer.

## Supplementary Materials

The following are available online at https://www.mdpi.com/1424-8247/13/12/484/s1, Figure S1: Linearity of the average of 3 calibrate curve; Figure S2: Apremilast chromatograms by HPLC; Figure S3: Rheogram of apremilast microemulsion (APR-ME) showing flow and viscosity curves at 25 °C; Figure S4: Transmission profiles of apremilast microemulsion after 1, 30, 60 and 90 days of production; Table S1: Standards of APR to analyze the linearity; Table S2: Precision inter-day; Table S3: Accuracy of the analytical method; Table S4: Robustness of the analytical method; and Table S5: Limit of detection (LOD) and limit of quantification (LOQ) of the analytical method.
