# Supplementary material for: Apremilast Microemulsion as Topical Therapy for Local Inflammation: Design, Characterization and Efficacy Evaluation
Source: Pharmaceuticals (Basel). 2020 Dec 21;13(12):484. doi: 10.3390/ph13120484 (PMC7767333; doi:10.3390/ph13120484)
Supplement: Supplementary file 1 [file pharmaceuticals-13-00484-s001.pdf]

Supplementary Materials

Table S1. Standards of APR to analyze the linearity.

Concentration (µg/ml)	Area (µV*sec) R1	R2	R3	Average Area (µV*sec)
1.25	144612	147653	150885	147717 ± 2561
2.5	281224	294306	300770	292100 ± 8131
5	578459	590112	604541	591037 ± 10668
10	1166899	1171264	1247082	1195081 ± 36813
25	2862244	2913060	3017704	2931003 ± 64722
50	5784489	5906320	6015409	5902072 ± 94321
100	11368977	11312239	12010818	11564011 ± 316788
200	23137955	23624478	24041635	23601356 ± 369288
r^2	0.9999	0.9996	1.000	

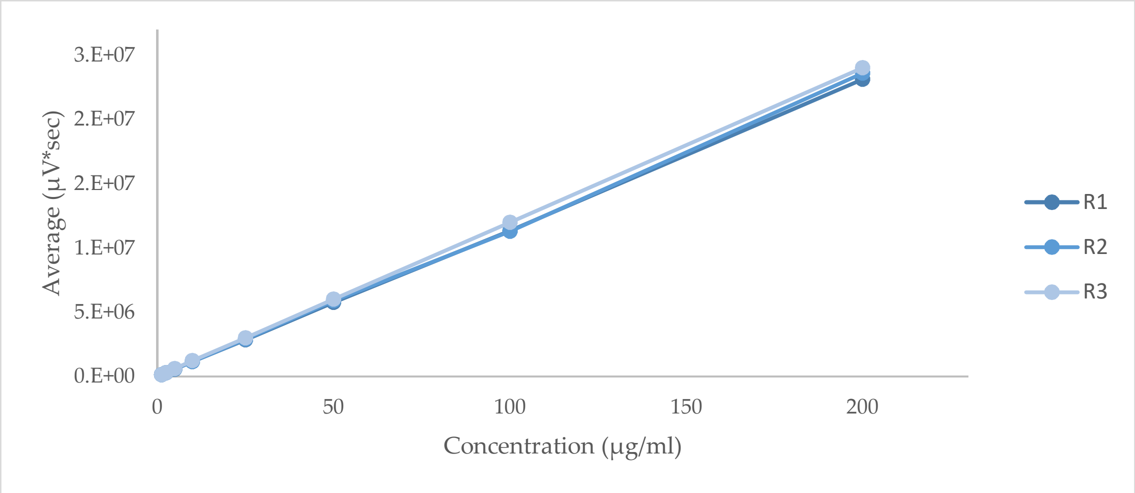

Figure S1. Linearity of the average of 3 calibrate curve. The test showed that deviation from linearity is not significant (p = 0.9068).

Table S2. Precision inter-day

Concentration (μg/ml)	Area (μV*sec)			Average (μV*sec)	SD	RSD%	Method Precision (%)
	Day 1	Day 2	Day 3				
1.25	144612	147653	150885	147717	3137	2.12	97.88
2.5	281224	294306	300770	292100	9958	3.41	96.59
5	578459	590112	604541	591037	13066	2.21	97.79
10	1166899	1171264	1247082	1195081	45086	3.77	96.23
25	2862244	2913060	3017704	2931003	79268	2.70	97.30
50	5784489	5906320	6015409	5902072	115519	1.96	98.04
100	11368977	11312239	12010818	11564011	387984	3.36	96.65
200	23137955	23624478	24041635	23601356	452284	1.92	98.08

SD = Deviation Standard; RSD = Relative Standard Deviation

Table S3. Accuracy of the analytical method.

Theoretical Concentration (μg/ml)	Real Concentration (μg/ml)			Average (μg/ml)	SD	Relative Error (%)	Method Accuracy (%)
	Day 1	Day 2	Day 3				
1.25	1.38	1.55	1.16	1.36	0.19	-8.36	108.36
2.5	2.56	2.80	2.41	2.59	0.19	-3.51	103.51
5	5.14	5.32	4.94	5.13	0.19	-2.57	102.57
10	10.24	10.27	10.29	10.26	0.02	-2.58	102.58
25	24.93	25.10	25.03	25.02	0.09	-0.08	100.08
50	50.25	50.59	49.98	50.28	0.31	-0.55	100.55
100	98.64	96.63	99.89	98.39	1.64	1.64	98.36
200	200.61	201.50	200.04	200.72	0.73	-0.36	100.36

SD = Deviation Standard; RE = Relative Error.

Table S4. Robustness of the analytical method: Variations of effect on the concentration *(v / v)* of the mobile phase.

Flux (ml/min)	Mobile Phase Concentration (v / v)	Average Retention Time (min)	SD
1	A: 60<br>B: 40	4.25	0.026
1	A: 70<br>B: 30	3.35	0.008
1	A: 80<br>B: 20	2.45	0.008

SD: Standard Deviation

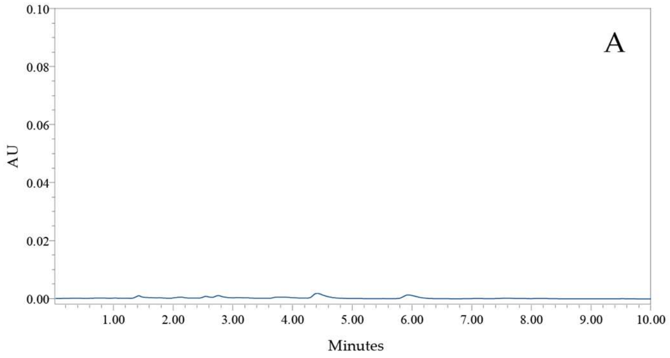

Peak Results								
Nº	Sample Name	Vial	Name	RT	Area (µV*sec)	Height	Amount	Unit
1	Blank control	3	Apremilast				Missing	mg/ml

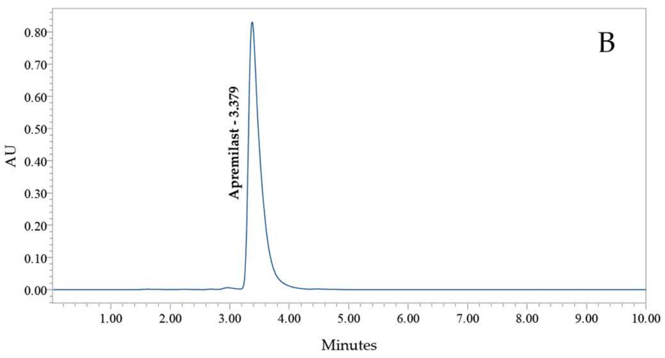

Peak Results								
Nº	Sample Name	Vial	Name	RT	Area (µV*sec)	Height	Amount	Unit
11	100µg/ml_APR	18	Apremilast	3.379	11629185	830052	0.1023	mg/ml

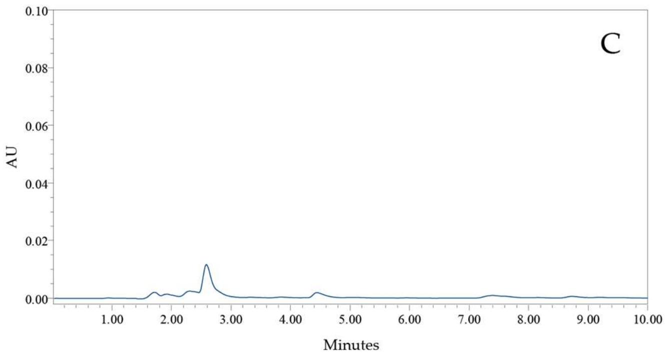

Peak Results								
Nº	Sample Name	Vial	Name	RT	Area (µV*sec)	Height	Amount	Unit
56	Blank skin	89	Apremilast				Missing	mg/ml

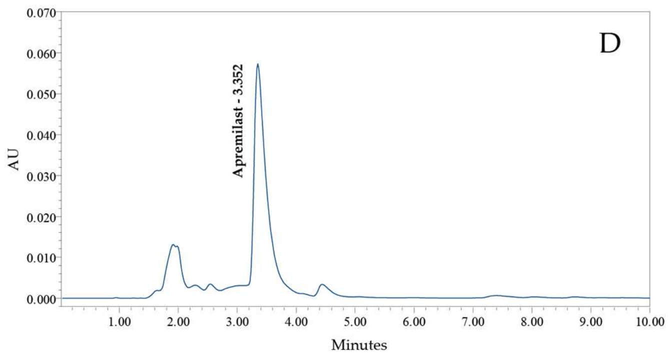

Peak Results								
Nº	Sample Name	Vial	Name	RT	Area (µV*sec)	Height	Amount	Unit
73	Qret_APR	25	Apremilast	3.352	773018	54764	0.0068	mg/ml

Figure S2. Apremilast chromatograms by HPLC. **(A)** Blank sample. **(B)** Apremilast Standard Sample 100 µg/mL. **(C)** Blank skin sample. **(D)** Apremilast extracted from human skin after the permeation study.

Table S5. Limit of detection (LOD) and limit of quantification (LOQ) of the analytical method.

	Average (µg/ml)	SD
**LOD**	1.13	1.04
**LOQ**	3.42	3.16

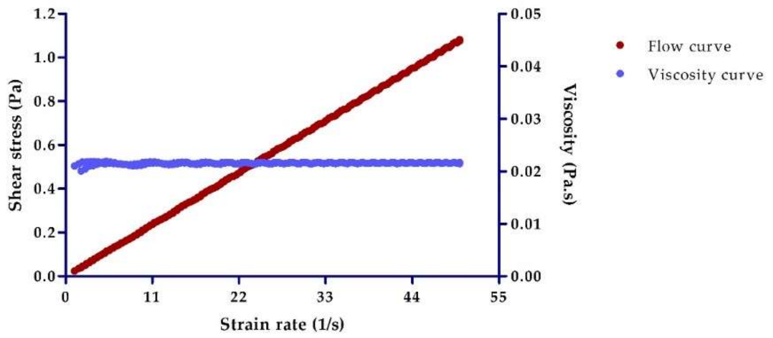

Figure S3. Rheogram of apremilast microemulsion (APR-ME) showing flow and viscosity curves at 25 °C.

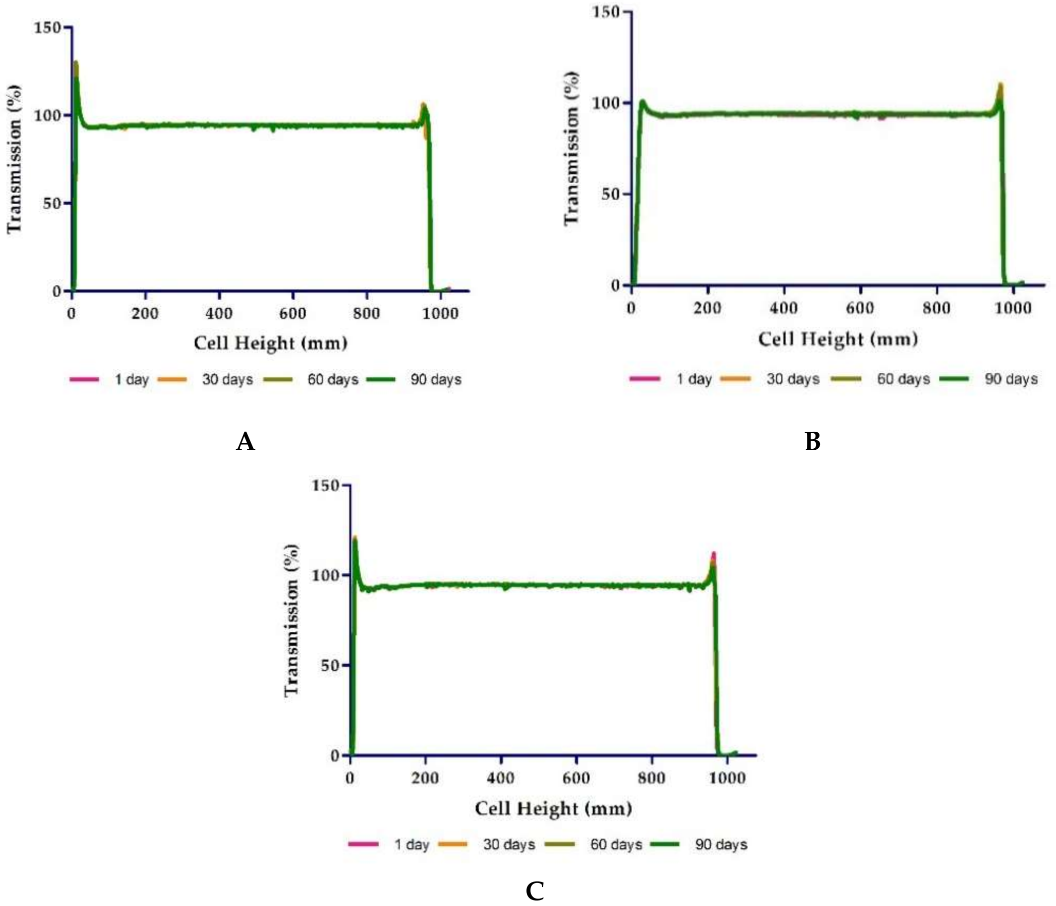

Figure S4. Transmission profiles of apremilast microemulsion after 1, 30, 60 and 90 days of production. **(A)** Storage 4 ± 1°C; **(B)** Storage 30 ± 2°C; and **(C)** Storage 40 ± 2°C.
